# Association of Methylated DNA Markers with High-Risk HPV Infections in Oral Site and Precancer Anal Lesions in HIV-Positive MSM

**DOI:** 10.3390/biomedicines12081838

**Published:** 2024-08-13

**Authors:** Silvia Pauciullo, Daniele Colombo, Verdiana Zulian, Roberta Sciamanna, Antonio Coppola, Alessandra Scarabello, Franca Del Nonno, Anna Rosa Garbuglia

**Affiliations:** 1 Laboratory of Virology, National Institute for Infectious Diseases “Lazzaro Spallanzani” (IRCCS), 00149 Rome, Italy; silvia.pauciullo@inmi.it (S.P.); verdiana.zulian@inmi.it (V.Z.); roberta.sciamanna@inmi.it (R.S.); antonio.coppola@inmi.it (A.C.); 2 Pathology Unit, National Institute for Infectious Diseases “Lazzaro Spallanzani” (IRCCS), 00149 Rome, Italy; daniele.colombo@inmi.it (D.C.); franca.delnonno@inmi.it (F.D.N.); 3 Clinical and Research Infectious Diseases Department, National Institute for Infectious Diseases “Lazzaro Spallanzani” (IRCCS), 00149 Rome, Italy; a.scarabello@inmi.it

**Keywords:** human papillomavirus, DNA methylation, biomarkers, high-grade anal intraepithelial neoplasia, oral cancer

## Abstract

Background: Human papillomavirus (HPV) infection is linked to several cancers, including anal and oral cancers. The incidence of anal cancer is particularly high among HIV-positive men who have sex with men (MSM). DNA methylation markers have shown promise as biomarkers for identifying precancerous lesions and cancer in HPV-infected individuals. The aim of this study was to investigate the correlation of DNA methylation with HPV infection in oral samples and the correlation of DNA methylation with lesion degree in the anal samples of HIV-positive MSM. Methods: This study investigated DNA methylation in oral and anal samples from HIV-positive MSM at the National Institute for Infectious Diseases (INMI) in Rome, Italy. Exfoliated oral epithelial cells and anal samples were collected and analyzed for 28 HPV genotypes using the Allplex 28 HPV assay. DNA methylation was assessed with the PrecursorM+ kit for oral samples and the AnoGyn kit for anal samples, focusing on the promoter regions of specific genes. Results: The study included 63 participants, with a median age of 49 and a median CD4+ count of 705 cells/µL. The oral samples showed HPV16 as the most common type, with 22% testing positive for DNA methylation. The anal samples exhibited HPV-related methylation changes linked to cytological lesions, with a 30% increase in the observed ddCt ratio. Significant differences were found in both ASCL1 and ZNF582 genes, particularly for HSIL*vs*NILM and HSIL*vs*LSIL lesions. Of the samples with an increased ddCt ratio, 80% were from patients over 35 years old, and multiple HPV infections were common. Conclusions: DNA methylation markers could be valuable in identifying high-risk HPV infections in oral samples and detecting potential precancerous lesions in anal samples. These markers may enhance the early detection and prevention strategies for HPV-related cancers in high-risk populations, with follow-up data indicating potential for monitoring lesion progression.

## 1. Introduction

Human papillomavirus (HPV) infection had been recognized as causal agent or various cancers beyond cervical cancer, including head and neck cancers (HNCs), and neoplasms of the anus, vagina, and penis with different prevalence [1]. In men, >90% of anal cancers are HPV-positive, particularly for HPV16 [2], with squamous cell carcinoma (SCC) being the most common malignant neoplasia affecting the anal canal [3,4]. Most HPV-related SCCs develop from dysplastic squamous precursor lesions, making early identification crucial for preventing malignant neoplasia. The incidence of anal cancer is significantly higher in high-risk populations, such as people living with HIV (PLWH), men who have sex with men (MSM), and women with a history of HPV-related cervical precancerous lesions, with a cancer rates 4 to 50 times higher compared to the general population [5,6]. In particular, MSM aged over 35 living with HIV have an anal cancer incidence ten times higher than the general population, with a prevalence of 54.6% for anal high-grade squamous intraepithelial lesions (HSILs) [7]. Among these, an estimated 10% of anal HSIL cases progress to anal cancer [8]. The HPV prevalence in HNCs varies, with HPV DNA positivity rates of 24.2% in oral cancers [9] and 30.1% in oral squamous cell carcinoma (OSCC) [10]. The prevention strategies for HPV-related cancers differ by tumour type. For anal cancer, screening programmes often include anal cytology and high-resolution anoscopy (HRA) with biopsies of suspected lesions, similarly to cervical screening for the early diagnosis and treatment of premalignant lesions [11]. However, the effectiveness of screening for reducing anal cancer incidence remains uncertain [12]. The Anal Cancer–HSIL Outcomes Research (ANCHOR) study recently showed that treating HSILs reduces the incidence of anal squamous cell carcinoma (ASCC) by 57% compared to no treatment with close monitoring [13] and earlier treatment could facilitate a reduction in the development of anal cancer. In contrast, no established screening strategies exist for oral and oropharyngeal cancers, despite their rising incidence in PLWH. Limited data are available on the predictors of oral HPV infections and related lesions, and their correlation with anal HPV infections in this at-risk group, and the utility of oral cytology and oroscopy in detecting oral cancer precursors, is unknown [11]. The natural history of HPV-caused oropharyngeal cancer is understudied, with no recognized precancerous lesions, thus making secondary prevention challenging. To optimize the prevention and screening for oral and anal HPV-related cancers, additional biomarkers, such as DNA methylation, could be valuable. DNA methylation suppresses the gene expression involved in cell cycle arrest and terminal differentiation in epithelial cells [14]. HPV can stimulate host DNA methylation by E6 and E7 proteins, enhancing the expression of the DNA methyltransferase (DNMT) [14,15], which increases DNA instability, leading to the oncogene expression and inactivation of tumour suppressor activity [16]. Higher DNA methylation levels in HPV-positive individuals have been noted across various HPV-related tumours [17]. Nakagawa et al. highlight distinct DNA methylation patterns in HPV-associated head and neck squamous cell carcinoma (HNSCC) compared to HPV-negative cases. Tumours without HPV integration exhibit high DNA methylation and better prognosis, whereas those with HPV integration show intermediate methylation and worse outcomes [17].

Altogether, these findings suggest that specific epigenetic changes could aid in developing targeted therapies and improving patient stratification.

The aims of this study were to describe the role of DNA methylation gene in oral samples infected with low-risk (LR) and high-risk (HR) HPV genotypes in a cohort of HIV-infected MSM who attended the Sexually Transmitted Disease Care Unit at INMI L Spallanzani. We also explored the host methylation correlation with anal HPV-correlated lesion in the same patients.

## 2. Materials and Methods

The study included HIV-positive MSM attending the Sexually Transmitted Disease Care Unit. HIV-positive MSM who resulted HPV positive in oral sample were consecutively included from September 2023 to April 2024. A DNA methylation test was performed both in oral and anal samples in each patient.

Specimen collection, processing, and DNA purification. Exfoliated oral epithelial cells were collected via oral rinse and gargle with mineral water, as previously described [18]. A physician also collected anal samples by using saline-moistened swabs [18]. For each patient, the following variables were recorded: age, CD4+ T cell count, actual viral load, and antiretroviral treatment. DNA was isolated from oral rinse and anal swabs using a magnetic bead-based automated platform QIASYMPPHONY (QIAGEN, Hilden, Germany). Purified DNA was evaluated for the presence of 28 HPV genotypes by the use of an Allplex 28 HPV SEEGENE real-time assay (SEEGENE, Seoul, Republic of Korea). HPV types were classified into non-oncogenic (low-risk, LR HPV6,11,26,40,42,43,44,54,61,70) and HR/potential carcinogenic (HPV16,18,26,31,33,35,39,45,51,52,53,56,58,59,68,69,73,82). Allplex HPV28 detection assays were performed using a CFX real-time thermocycler (Bio-Rad, Hercules, CA, USA). A Cq cut-off value of 42.00 was used for positivity for all HPV genotypes.

Cytology. The anal and oral samples were also used to perform cytology analysis. The cytology results were classified as normal, atypical squamous cells of uncertain significance (ASCUS), atypical squamous cells cannot exclude HSIL (ASC-H), low grade squamous intraepithelial lesion (LSIL), high-grade squamous lesion (HSIL), and squamous cell carcinoma (SCC), in accordance with the Bethesda classification system [19].

DNA methylation marker analysis. The DNA methylation analysis was carried out with two different commercially available diagnostic kits: the CE-IVD PrecursorM+ kit (distributed by Fujirebio Europe, Gent, Belgium) for oral samples, using this type of application outside of the manufacturer’s indications; the RUO AnoGyn kit (distributed by Fujirebio Europe), for the analysis carried out on anal samples. The PreCursorM+ Assay analyses in a multiplex methylation-specific real-time PCR the methylation of the promoter regions of FAM19A4 and miR124-2, and the ACTB gene, encoding for beta-actin, as a reference gene. Instead, the AnoGyn kit analyzes, in a multiplex methylation-specific real-time PCR, the methylation of the promoter regions of ASCL1 and ZNF582 and a reference gene (ACTB). Briefly, genomic DNA was isolated as described above and its concentration measured with the Qubit™ 4 Fluorometer Instrument (Thermo Fisher, Waltham, MA, USA) using the Qubit 1X dsDNA BR Assay Kit (Thermo Fisher), an assay designed to measure the genomic DNA concentration in a range of 4–4000 ng. In the bisulfite reaction up to 200 ng/45 μL, isolated genomic DNA was converted using the EZ DNA Methylation Kit (Zymo Research Europe, Freiburg im Breisgau, Germany) following the manufacturer’s instructions.

To perform both PreCursorM+ and AnoGyn Test, 17.5 μL of the respective ready-to-use real-time PCR Master Mix and 2.5 μL of DNA-positive and -negative controls were added to PCR instrument tubes. The multiplex PCR was run on a MIC IVD instrument (Bio Molecular Solutions, Upper Coomera, QLD, Australia).

The samples were scored as valid when the Ct value of ACTB was ≤26.4 for the PreCursorM+ and when the Ct value of ACTB was ≤30 for the AnoGyn, according to manufacturer instructions. For both kits, the ΔΔCt values for each target gene were calculated with the following formula: ΔΔCt target gene = (Ct target gene sample − Ct ACTB sample) − (Ct target gene calibrator − Ct ACTB calibrator). For the PrecursorM+ analysis, oral samples were considered hypermethylation-positive if at least one of the methylation marker genes had a ΔΔCt below the cut-off that the manufacturer provide for analysis of cervical self-collected samples (10.36 for FAM19A4 and 6.5 for miR124-2). These cut-offs were applied empirically, as there is currently no supporting literature on samples taken from the oral cavity. For the AnoGyn Analysis, given that the kit is available on the market as RUO, there are no pre-established cut-offs that can be applied even empirically. The methylation level of a target in anal samples is expressed as the ‘ΔΔCt ratio’ (2^−ΔΔCt^ * 100). The interpretation was performed using an incremental increasing scale starting at 0, in which 0 indicates no methylation, and increasing numbers indicate higher methylation levels.

Statistical analyses. Statistical analyses and graphical representations were performed using GraphPad Prism version 9 (GraphPad Software, La Jolla, CA, USA). A significance level of *p* < 0.05 was chosen for all statistical tests.

The categorical variables were given as numbers and percentages, and the continuous variables as median values and interquartile range (IQR). Fisher’s exact test was used to examine the association between methylation positivity vs. multi-genotype HPV infection. An unpaired Mann–Whitney test was used to compare the ddCt ratio of ASCL1 and ZNF582 genes between the several lesion grades (NILM, ASCUS/ASC-H, LSIL, and HSIL).

## 3. Results

**Figure 1:**
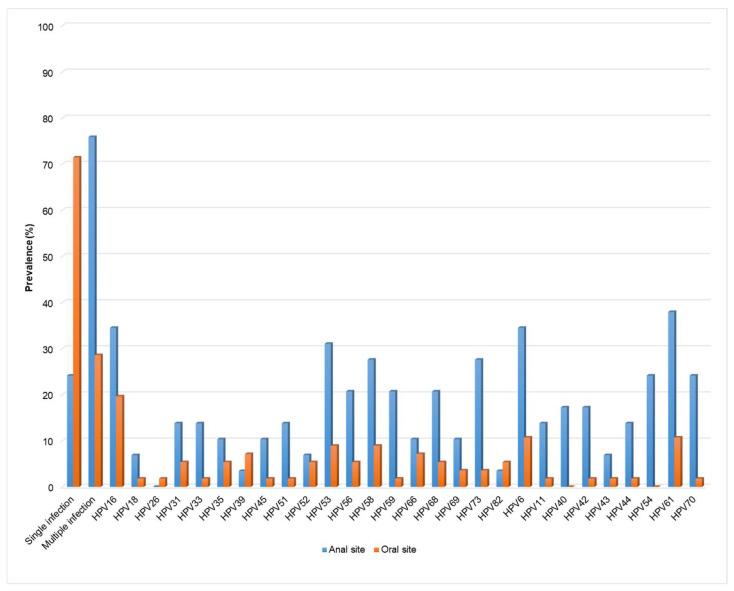
Distribution of the HPV genotypes detected in oral and anal sites. HPV, human papillomavirus.

The characteristics of the 63 participants included in the study are shown in Table 1. All participants were receiving HAART with a current median CD4+ T cell count of 705 cells/μL and a median age of 49 years (Table 1); the majority of participants had an HIV RNA viral load <30 copies/mL (80.9%). Figure 1 shows the distribution of the detected HPV genotypes.

### 3.1. Oral Samples

Concerning oral site (Table 2), a total of 50 samples were analyzed with the PrecursorM+ assay, whereas 13 specimens were excluded because the DNA sample volume was not sufficient for the bisulfite conversion step (*n* = 11) or because ACTB gave no amplification signal (*n* = 2). A total of 44 specimens harboured at least one HR-HPV or probable carcinogenic HPV types, with the HPV16 being the most represented (*n* = 10). Multiple HPV infections were observed in 15 samples. No neoplastic lesions were observed in any oral samples.

Among 50 oral samples, 11 (22.00%) were considered positive for methylation. The prevalence of methylation positivity in these samples was 9.09% for LR-HPV and 90.91% for HR-HPV. However, the differences in methylation prevalence compared to the methylation-negative samples were not statistically significant (*p* = 0.66).

In samples considered as positive for DNA methylation, the median age was 57 years, and the CD4+ T cell count was 747 cells/µL (618.5–997.5), which did not differ from the median value (*p* > 0.05). Additionally, the exact Fisher’s test analysis highlighted no statistical significance (*p* > 0.9999) for oral samples in relation to the association between methylation positivity and multi-genotype HPV infection.

Four patients also presented follow-up samples; thus, overall, we analyzed 54 oral specimens. The ages of these patients were as follows: 56 years (y) (FU14), 49 y (FU31), and 29 y (FU34 and FU44). Pt 14, who showed DNA methylation on the FAM19A4 gene in both samples, was infected with HPV61, whereas the other three couple samples with follow-up (Pt 14-FU14; Pt 31-FU31; Pt 34-FU34; Pt 44-FU44) showed no methylation in both time points. A second HPV61-positive patient (Pt 41) was positive for DNA methylation. No oral neoplastic lesions were observed among any patients.

### 3.2. Anal Samples

**Figure 2:**
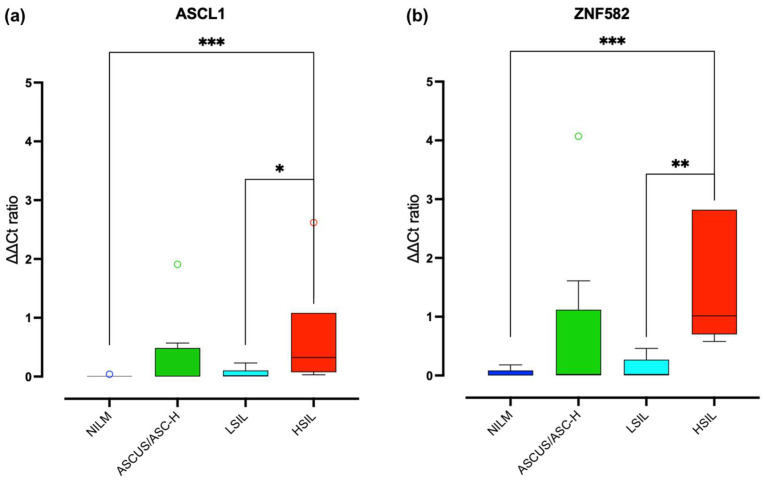
Differential DNA methylation of ASCL1 (**a**) and ZNF582 (**b**) genes in anal samples. Data are expressed as ddCt ratio, as described in Section 2. NILM, negative for intraepithelial lesion or malignancy; ASCUS, atypical squamous cells of undetermined significance; LSIL, low-grade squamous intraepithelial lesion; HSIL, high-grade squamous intraepithelial lesion. * = *p* < 0.05; ** = *p* < 0.01; *** = *p* < 0.001.

Among the 63 anal specimens analyzed for DNA methylation with the AnoGyn kit, 33 were excluded because the amount of DNA was not sufficient for the bisulfite conversion step (*n* = 20) or because ACTB gave no amplification signal (*n* = 13). A total of 35 specimens harboured at least one HR-HPV or probable carcinogenic HPV types. HPV multiple infection was observed in 33 samples. The results obtained are shown in Figure 2. The samples were divided by cytological category: 11 NILM (absence of lesions), 4 ASCUS and 4 ASC-H (grouped into a single category), 5 LSIL, and 6 HSIL; it is also possible to observe how each cytological category presents a different distribution of the ddCt ratio, which increases as the lesion is observed. An increased ddCt ratio was observed in 30% of the anal samples analyzed (*n* = 10); 10% of these samples were associated with LR-HPV (*n* = 1), 90% were associated with HR-HPV (*n* = 9), and 50% exhibited an HSIL (*n* = 5), with methylation that seems more pronounced for the ZNF582 gene than for the ASCL1 gene.

In addition, there were statistically significant differences in the ddCt ratio as follows: (1) ASCL1 gene in HSIL vs. NILM (*p* = 0.0007) and HSIL vs. LSIL (*p* = 0.026); (2) ZNF582 gene in HSIL vs. NILM (*p* = 0.001) and HSIL vs. LSIL (*p* = 0.0043).

Furthermore, 80% of the samples with an increased ddCt ratio showed the presence of multiple infections (*n* = 8).

The median age of samples with an increased ddCt ratio was 50.5 versus 47 years in samples with a ddCt ratio near to zero, while the median CD4+ T cell count was 578 (450.0–782.5) and 692 (157–749) cells/μL, respectively. Finally, among the samples with an increased ddCt ratio, 90% (*n* = 9) were from patients aged over 35 years, while 10% (*n* = 1) were associated with patients aged under 35 years.

## 4. Discussion

The findings shown in the present study, though preliminary, offer valuable insights into the potential use of DNA methylation testing in preventing HPV-related oral and pharyngeal tumours. Among our patients, all but two who tested positive for DNA methylation were infected with HR-HPV in oral samples. Interestingly, HPV61, typically considered low-risk for genital and anal infections, was found in samples of HNC and anal carcinoma [20,21]. Further evidence is needed to clarify HPV61’s oncogenic role, particularly in the oropharyngeal region. However, during the follow-up, Pt 14 also tested positive for HR-HPV52 (Table 2), which may have facilitated the persistence of the hypermethylation status.

It is important to note that our observation of four follow-up samples confirmed previous findings, suggesting that HPV transformation in the oral cavity could occur gradually over at least six months following the initial detection. Indeed, no significant changes in methylation status were observed in any six-month follow-up, and positivity for DNA methylation (Pt 14) was confirmed in subsequent follow-up samples. There was no correlation between oral and anal methylation status. At the anal level, methylation status could serve as a parameter for determining the intervals for cytological testing or high-resolution anoscopy (HRA) in cases of HR-HPV presence, and this biomarker could prove particularly useful in settings where HRA infrastructure is limited for referring patients with abnormal screening results. Additionally, it may provide a further interpretation of ASCUS cases where abnormal cytological findings do not offer sufficient indications of clinical impact. Our data confirm those reported in the study by Rozemeijer et al., wherein a greater degree of methylation was observed in subjects with HSIL compared to those with LSIL and ASCUS [22], and in the work by Vasavada, who analyzed the DNA methylation of different genes: ASCL1 and FMN2 [23]. Furthermore, over 90% of the patients with an increased ddCt ratio in anal samples were aged >35 years. This observation supports the IANS recommendation to initiate anal cancer screening for HIV-positive MSM aged >35 years [24]. One of the limitations of our study is the choice of cut-off values. Specifically, the cut-offs used in our analysis were not originally defined for DNA methylation in the sites investigated in our preliminary study. Instead, we used cut-off values that were established for the uterine cervix. To provide new insights on cut-off definition, additional patients will be enrolled in a longitudinal follow-up study to verify the use of these biomarkers in the prevention of HPV-related SCC in the oral cavity.

Consequently, one of our future objectives is to implement and clearly define cut-offs that can be used according to the specific site being analyzed; alternatively, the cut-offs may be applicable to both anatomical sites.

Other limitations of this study include the small number of patients and follow-up samples from the oral cavity. However, the association of all oral methylation-positive samples with HR-HPV is encouraging for conducting longitudinal studies that incorporate DNA methylation testing into screening protocols. For anal samples, associating DNA methylation results with HRA findings may provide necessary insights into the relevance of this new test as a predictor of HPV oncogenic transformation.

## Figures and Tables

**Table 1:** Baseline characteristics in persons included in the study.

Characteristics	
Median age, years (IQR)	49 (38.5–68.0)
Median CD4+ T cell count (cells/μL) (IQR)	705 (486.0–847.0)
HIV viral load—*N* (%)	
Undetectable	51 (80.9)
Detectable	2 (3.2)
Unknown	10 (15.9)
**Lesion grade**	
Oral—*N* (%)	
NILM	30 (47.6)
ASCUS	33 (52.4)
Anal—*N* (%)	
NILM	19 (44.2)
ASCUS	11 (25.6)
LSIL	7 (16.2)
HSIL	6 (14.0)

Abbreviations: NILM, negative for intraepithelial lesion or malignancy; ASCUS, atypical squamous cells of undetermined significance; LSIL, low-grade squamous intraepithelial lesion; HSIL, high-grade squamous intraepithelial lesion; IQR, interquartile range (IQR1-IQR3). Viral load is categorized as undetectable for individuals with <30 copies/mL and detectable otherwise per clinical guidelines.

**Table 2:** Relative DNA methylation (ddCt values) of FAM19A4 and mir124-2 genes in oral samples.

*Baseline*	*ddCtFAM19A4*	*ddCtmir124-2*	*Result*	*Cytology*	*Genotype*
*Pt 1*	11.67389416	-	Negative	ASCUS	**HPV16**
*Pt 2*	9.453338358	11.4266975	Positive	NILM	**HPV82, 39**
*Pt 3*	11.33928662	14.21413224	Negative	ASCUS	**HPV68**
*Pt 4*	9.870100045	12.73096119	Positive	ASCUS	**HPV16**
*Pt 5*	9.454067616	-	Positive	ASCUS	**HPV16**
*Pt 6*	11.8962865	11.98879913	Negative	ASCUS	**HPV16**
*Pt 7*	10.50086255	9.409393794	Negative	ASCUS	HPV6
*Pt 8*	12.05459854	14.7422688	Negative	NILM	HPV42
*Pt 9*	11.33288572	11.13614887	Negative	ASCUS	**HPV18**
*Pt 10*	10.9173894	10.39343708	Negative	ASCUS	**HPV52**
*Pt 11*	12.17853289	10.84083335	Negative	NILM	**HPV56,58**
*Pt 12*	11.62954436	9.511607074	Negative	ASCUS	**HPV35**,6
*Pt 13*	12.29596614	9.472156942	Negative	ASCUS	**HPV51**
*Pt 14*	9.884411623	10.75950655	Positive	NILM	HPV61
*Pt 15*	9.87684043	9.049550437	Positive	ASCUS	**HPV53**
*Pt 16*	13.2953954	10.30919426	Negative	ASCUS	**HPV68**
*Pt 17*	-	10.05682617	Negative	ASCUS	HPV6
*Pt 18*	10.97464054	9.605091426	Negative	ASCUS	**HPV31,53**
*Pt 19*	10.65275917	10.93071802	Negative	NILM	**HPV31,69**
*Pt 20*	12.34449267	10.59708123	Negative	NILM	**HPV69**
*Pt 21*	13.10534787	11.00575345	Negative	ASCUS	**HPV26**,70
*Pt 22*	12.91008603	11.03519112	Negative	ASCUS	HPV44
*Pt 23*	11.68822231	10.68655588	Negative	ASCUS	**HPV16,66**
*Pt 24*	10.08326187	9.106563818	Positive	ASCUS	**HPV45**
*Pt 25*	9.470165301	9.55831486	Positive	NILM	**HPV33,35**,43
*Pt 26*	14.22511213	11.03932987	Negative	NILM	**HPV16**
*Pt 27*	7.057501302	7.987395465	Positive	NILM	**HPV53**
*Pt 28*	10.88859044	10.11865056	Negative	NILM	**HPV31**
*Pt 29*	11.43293716	9.061973272	Negative	NILM	**HPV66**
*Pt 30*	8.37381873	8.626123558	Positive	NILM	**HPV16**
*Pt 31*	-	-	Negative	ASCUS	**HPV56,58,59,66**
*Pt 32*	11.29022259	9.572790052	Negative	NILM	**HPV82**
*Pt 33*	13.57058146	10.62152985	Negative	ASCUS	HPV11,**73**
*Pt 34*	14.19290116	10.47430333	Negative	ASCUS	HPV6
*Pt 35*	-	9.556213112	Negative	NILM	**HPV35**,61
*Pt 36*	-	13.91914873	Negative	NILM	**HPV68**
*Pt 37*	11.44630628	9.389119266	Negative	NILM	**HPV53,82**
*Pt 38*	10.78752532	8.67125565	Negative	ASCUS	**HPV16**
*Pt 39*	12.70923461	10.12196678	Negative	ASCUS	HPV6
*Pt 40*	8.7231532	7.957266548	Positive	ASCUS	**HPV45**
*Pt 41*	10.11	9.67	Positive	NILM	HPV61
*Pt 42*	10.96	8.78	Negative	ASCUS	HPV61
*Pt 43*	12.66397256	-	Negative	NILM	**HPV39,66**
*Pt 44*	-	10.10600421	Negative	NILM	**HPV16**
*Pt 45*	10.99237382	11.43245412	Negative	ASCUS	**HPV58**
*Pt 46*	11.69903698	9.075850661	Negative	NILM	**HPV52,58**
*Pt 47*	-	9.520737204	Negative	ASCUS	**HPV16**
*Pt 48*	10.8267296	10.43430522	Negative	NILM	**HPV58,73**
*Pt 49*	11.41951603	13.13531765	Negative	ASCUS	HPV61
*Pt 50*	11.61742284	-	Negative	ASCUS	**HPV53**
***Follow-Up***	***ddCtFAM19A4***	***ddCtmir124-2***	***Result***	***Cytology***	***Genotype***
*FU14*	10.20468041	10.07825271	Positive	ASCUS	**HPV52**, HPV61
*FU31*	-	-	Negative	NILM	**HPV39**
*FU34*	13.00001938	10.41566436	Negative	NILM	HPV6
*FU44*	-	-	Negative	ASCUS	**HPV16**

Considering the cut-off values used and described in Section 2, we consider a sample “Positive” where the ddCt value of at least one of the two genes is lower than the cut-offs, and “Negative” where the ddCt values of both genes is higher than the cut-off. Abbreviations: NILM, negative for intraepithelial lesion or malignancy; ASCUS, atypical squamous cells of undetermined significance. High-risk HPV genotypes are indicated in bold. FU, follow-up of some enrolled patients.

## Data Availability

All data generated or analyzed during this study are included in this published article.

## Abbreviations

ACTB	β-actin
AIN	anal intraepithelial neoplasia
ASC-H	atypical squamous cells cannot exclude HSIL
ASCL1	achaete-scute family bHLH transcription factor 1
ASCUS	atypical squamous cells of undetermined significance
CI	confidence interval
Cq	quantification cycle
DNMT	DNA methyltransferase
FAM19A4	family with sequence similarity 19 member A4
FMN2	formin 2
HAART	highly active antiretroviral therapy
HGAIN	high-grade anal intraepithelial neoplasia
HIV	human immunodeficiency virus
HNC	head and neck cancer
HPV	human papillomavirus
HRA	high-resolution anoscopy
HR-HPV	high-risk human papillomavirus
HSIL	high-grade squamous intraepithelial lesion
IANS	International Anal Neoplasia Society
IQR	interquartile range
LR-HPV	low-risk human papillomavirus
LSIL	low-grade squamous intraepithelial lesion
Mir124-2	microRNA 124-2
MSM	men who have sex with men
NILM	negative for intraepithelial lesion or malignancy
PCR	polymerase chain reaction
PLWH	people living with HIV
RUO	research use only
SCC	squamous cell carcinoma
ZNF582	zinc finger protein 582
